# Docetaxel, Cisplatin, and 5‐Fluorouracil as perioperative chemotherapy compared with surgery alone for resectable gastroesophageal adenocarcinoma

**DOI:** 10.1002/cam4.885

**Published:** 2016-10-11

**Authors:** Frédéric Fiteni, Sophie Paget‐Bailly, Mathieu Messager, Thierry N'Guyen, Zaher Lakkis, Pierre Mathieu, Najib Lamfichekh, Alain Picard, Bilell Benzidane, Denis Cléau, Franck Bonnetain, Christophe Borg, Christophe Mariette, Stefano Kim

**Affiliations:** ^1^ Department of Medical OncologyUniversity Hospital of BesançonBesançonFrance; ^2^ Methodology and Quality of Life in Oncology UnitUniversity Hospital of BesançonBesançonFrance; ^3^ Lille University HospitalDepartment of Digestive SurgeryLilleFrance; ^4^ FREGAT (French Esophageal and Gastric Tumour) working groupParisFrance; ^5^ Department of Digestive Surgery and Liver TransplantationUniversity Hospital of BesançonBesançonFrance; ^6^ Department of SurgeryNord Franche Comté HospitalMontbeliardFrance; ^7^ Department of SurgeryNord Franche Comté HospitalBelfortFrance; ^8^ Department of Oncology and RadiotherapyNord Franche Comté HospitalMontbeliardFrance; ^9^ Department of GastroenterologyHospital of VesoulVesoulFrance; ^10^ University of Franche‐ComtéBesançonEA 3181France; ^11^ Unit 1098INSERMUniversity of Franche‐ComtéBesançonFrance; ^12^ Clinical Investigational CenterUniversity Hospital of BesançonCIC‐1431France

**Keywords:** Chemotherapy, docetaxel, gastric cancer, neoadjuvant, surgery

## Abstract

Docetaxel, cisplatin, and 5‐fluorouracil (DCF) significantly improved overall survival in metastatic gastroesophageal adenocarcinoma (GEA). The aim of this study was to assess efficacy of DCF regimen as perioperative chemotherapy compared with surgery alone in patients with resectable GEA. We identified 789 patients who underwent surgery alone and 62 patients who received at least one cycle of DCF regimen consisting of docetaxel (75 mg/m^2^ on day 1), cisplatin (75 mg/m^2^ on day 1), and 5‐fluorouracil (750 mg/m^2^/day on continuous perfusion on days 1 to 5), every 3 weeks. Overall survival was compared using Cox proportional hazards regression model with adjustments for confounding factors provided by two propensity score methods: inverse probability of treatment weighting (IPTW) and matched‐pair analysis. In Cox multivariate analysis weighted by IPTW, DCF group was associated with favorable overall survival (OS) compared with the surgery group (HR = 0.59; 95% CI, 0.45–0.78; *P* = 0.0003). For the matched‐pair analysis (comparing 41 patients for each group with the same baseline characteristics), median OS was 22 months and 57 months for the surgery group and DCF group, respectively (log‐rank *P* = 0.0011). In Cox multivariate analysis, DCF group was associated with favorable OS compared with the surgery group (HR = 0.29; 95% IC, 0.14–0.64; *P* = 0.0019). In the matched‐pair population, major complications (Dindo‐Clavien grade 3–5) arose in six patients (14.63%) in the DCF group and seven patients (17.07%) in the surgery group (*P* = 1). Perioperative DCF chemotherapy is superior to surgery alone in terms of OS. A randomized phase III trial should compare DCF to standard perioperative regimens.

## Introduction

Perioperative chemotherapy significantly increased the median overall survival (OS) and complete resection rate (R0) over surgery alone for resectable gastroesophageal adenocarcinoma (GEA) patients. In 2006, the randomized phase III MAGIC study compared three preoperative and three postoperative cycles of intravenous epirubicin, cisplatin, continuous infusion fluorouracil combination (ECF), versus surgery alone and reported an improvement in OS (hazard ratio (HR), 0.75; 95% CI, 0.60–0.93; *P* = 0.0009) and disease‐free survival (DFS) (HR for progression, 0.66; 95% CI, 0.53–0.81; *P* < 0.001) for the chemotherapy group 1. In 2011, Ychou et al. demonstrated that perioperative chemotherapy using fluorouracil plus cisplatin (CF) significantly increased OS (HR, 0.69; 95% CI, 0.50–0.95; *P* = 0.02) and DFS (HR, 0.65; 95% CI, 0.48–0.89; *P* = 0.003) 2. Despite these encouraging results, the long‐term outcome remains dismal, with less than 40% of patients alive at 5 years. These two trials (MAGIC and FNCLCC 94012 FFCD 9703) were the first studies to demonstrate better survival rates with a perioperative systemic approach for the treatment of localized GEA. The meta‐analysis by Li et al. confirmed the benefit of neoadjuvant or perioperative chemotherapy in terms of survival rate 3, and neoadjuvant chemotherapy is now considered as standard treatment for resectable GEA in Europe.

At the metastatic setting, the V325 study demonstrated that docetaxel, cisplatin, and 5‐fluorouracil (DCF) significantly improved OS, time to progression, and quality of life over CF regimen 4.

At the preoperative setting, encouraging results were observed with DCF in a phase II trial including 43 patients. Surgery was carried out in 95% of patients, 95% had R0 resection and 9% had a pathologic complete response (pCR). Three‐year overall survival was 60%. No surgical mortality was observed in this study 5.

To date, perioperative DCF was not compared to surgery alone. Thus, to gain insight into the relative efficacy of DCF regimen, the aim of this study was to assess efficacy of DCF regimen as perioperative chemotherapy compared with surgery alone in a large multicenter comparative cohort of patients with resectable GEA.

## Patients and Methods

### Patient selection

Two French databases, including consecutive GEA patients in a multicentric setting, were used: the retrospective national survey conducted at 19 French surgical centers between January 1997 and January 2010, and a retrospective regional Franche‐Comté survey in 5 surgical centers, not included in the first survey, between January 1999 and December 2012.

**Figure 1:**
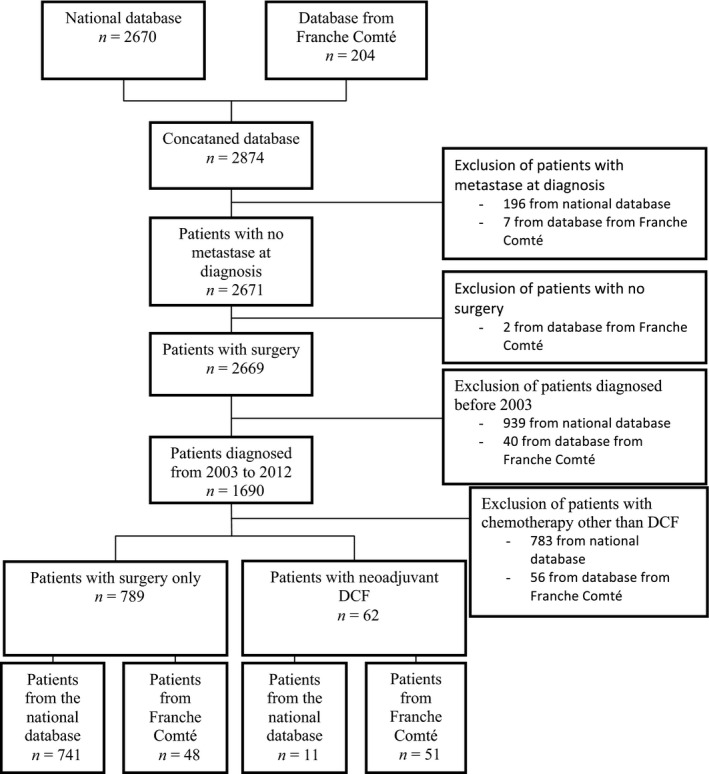
Flow chart of the population study.

Main inclusion criteria were as follows: resectable GEA (of the lower third of the esophagus or gastroesophageal junction or stomach), a proven histology of gastric adenocarcinoma, and absence of metastases. We identified patients who underwent surgery alone and those who received at least one cycle of DCF regimen consisted of docetaxel (75 mg/m^2^ on day 1), cisplatin (75 mg/m^2^ on day 1), and 5‐fluorouracil (750 mg/m^2^/day on continuous perfusion on days 1–5), every 3 weeks. No patient received DCF regimen before 2003, so patients who underwent a surgery before 2003 were excluded. From a total of 2874 patients, 851 patients fulfilled our eligibility criteria (Fig. 1).

### Study analysis

We used clinical records to obtain at baseline the gender, age at diagnosis, tumor localization, signed ring cell histology, and clinical stage (by American Joint Committee on Cancer classification version 6). We also obtained the type of surgery approach, the extension of lymph node dissection, respectability and metastases at surgery, pathological stage, and pathological complete resection characteristic. We used Clavien‐Dindo classification to grade surgical complications.

### Statistical analysis

Qualitative variables were described using frequency and percentage with 95% CI, and continuous variables were described using mean (SD) and median (Min‐Max). The differences in baseline characteristics between groups were tested using Fisher exact test or Student *t* test for categorical and continuous variables, respectively.

Propensity score analysis adjusts for the bias induced by nonrandom treatment assignment by comparing patients who had a similar likelihood of receiving a treatment but who received different treatments 6. For this analysis, we used logistic regression to predict the likelihood that a given patient would receive treatment with DCF. In order to take into account all baseline covariates in a nonparsimonious way, the multivariate model included the following variables: age, gender, site of tumor, tumor stage (cT) (T0 + T1 vs. T2 + T3 vs. T4), nodal stage (cN) (N0 vs. N+), signet ring cell, and year of diagnosis (≤2006, 2006–2009, and ≥2009). The Hosmer and Lemeshow goodness‐of‐fit statistics and the area under the ROC curve were calculated to evaluate the adequacy of the model.

The primary outcome was OS, defined as time interval between start of preoperative treatment and death from any cause for patients who received DCF and time interval between surgery and death of all causes for patients who underwent surgery only. The secondary outcome was to assess compliance of DCF regimen.

We estimated the effect of treatment on survival using the following two approaches: matching and weighting by inverse probability of treatment (IPTW). OS was estimated using Kaplan‐Meier estimation and described by median and 95% CI.

For the matched‐pair analysis, we matched each patient who received DCF with one who received surgery alone using caliper method with no replacement, with a caliper of 0.2 and a ratio of 1:1. To compare the groups, log‐rank test, and univariate and multivariate Cox models were performed.

For the IPTW analysis, in the univariate and multivariate Cox models, patients who received DCF were weighted by 1/propensity score, whereas patients who underwent surgery only were weighted by 1/(1‐propensity score).

In both matched‐pair and IPTW analyses, variables associated with OS in univariate analyses with a significance level of *P* < 0.20 were included in multivariate analysis.

All of the tests were two sided, and *P* < 0.05 was regarded as significant. The analyses were conducted using SAS 9.3 (SAS, Cary, NC).

## Results

### Patients' characteristics

Among the 851 patients included, 789 were treated with surgery alone and 62 patients received DCF perioperative chemotherapy (Fig. 1). Patients' characteristics are summarized in Table 1.

**Table 1:** Patient characteristics in the observational dataset and in patients who were matched by a propensity score

Characteristics	Patients with surgery alone		DCF Patients		*P* value^a^	Patients with surgery only^b^		DCF Patients^b^		*P* value^a^
	*n* = 789		*n* = 62			*n* = 41		*n* = 41		
	*n*	%	*n*	%		*n*	%	*n*	%	
Age					<0.0001					0.6281
<=65	234	29.66	44	70.97		19	46.34	24	58.54	
65–75	198	25.10	12	19.35		14	34.15	11	26.83	
>75	357	42.25	6	9.68		8	19.51	6	14.63	
Gender					0.1650					0.4410
Men	511	64.77	46	74.19		33	80.49	29	70.73	
Women	278	35.23	16	25.81		8	19.51	12	29.27	
Localization					<0.0001					1
Gastroesophageal junction and lower third of esophagus	173	21.93	31	50.00		21	51.22	21	51.22	
Stomach	616	78.07	31	50.00		20	48.78	20	48.78	
Signet ring cell					0.2807					1
Yes	49	6.22	6	9.68		3	7.32	4	9.76	
No	739	93.78	56	90.32		38	92.68	37	90.24	
Missing	1		0							
cT					<0.0001					0.8951
T0	9	1.88	0	0		0	0	0	0	
T1	141	29.38	2	3.33		1	2.44	2	4.88	
T2	162	33.75	19	31.67		17	41.46	15	36.59	
T3	159	33.13	37	61.67		22	53.66	23	56.10	
T4	9	1.88	1	1.67		1	2.44	0	0	
T4a	0	0	1	1.67		0	0	1	2.44	
Missing	309		2							
cN					<0.0001					0.6528
N0	393	69.19	21	34.43		18	43.90	15	36.59	
N+	175	30.81	40	65.57		23	56.10	26	63.41	
Missing	221		1							
Year of diagnosis					<0.0001					1
≤2006	341	48.99	4	6.56		2	4.88	3	7.32	
2006–2006	335	48.13	37	60.66		35	85.37	34	82.93	
>2009	20	2.87	20	32.79		4	9.76	4	9.76	
Missing	93		1							

DCF, Docetaxel, cisplatin, and 5‐fluorouracil

a Derived from Fisher test.

b Matched population.

In the multiple logistic regression analysis, younger age, esogastric junction and lower third esophagus location of tumor, more advanced stage, and later year of diagnosis were associated with the decision to use DCF regimen (Table S1). The AUC was equal to 0.93 and *P* value of Hosmer‐Lemeshow test was equal to 0.52, showing a good adequacy of the model.

For the matched‐pair analysis, 41 patients treated with DCF regimen and 41 patients treated with surgery only were matched based on their propensity score. This analysis eliminated the differences seen in the larger cohort (Table 1).

### Survival analysis

For the IPTW analysis, in the Cox multivariate analysis, the DCF group was associated with a favorable OS compared with the surgery group (HR=0.59; 95% CI, 0.45–0.78; *P* = 0.0003) (Table 2). The other variables associated with favorable OS were as follows: younger age, gastric location of tumor, adenocarcinoma histology, lower cT stage, and later year of diagnosis (Table 2).

**Table 2:** Cox regression for the IPTW analysis (*n* = 464)

		Univariate Cox analysis			Multivariate Cox analysis		
Parameters		HR	IC95%	*P*	HR	IC95%	*P*
Treatment	Surgery alone	1		<0.0001	1		0.0003
	DCF	0.602	0.474–0.763		0.590	0.445–0.784	
Age	≤55	1		<0.0001	1		<0.0001
	55–65	2.711	2.000–3.675		2.878	2.094–3.955	
	>65	1.628	1.199–2.211		1.898	1.373–2.623	
Gender	Men	1		0.7400			
	Women	1.049	0.792–1.388				
Localization	Gastroesophageal junction and lower third of esophagus	1		0.0031	1		<0.0001
	stomach	0.709	0.565–0.890		0.597	0.467–0.765	
Signet ring cell	No	1		0.0047	1		0.0068
	Yes	1.792	1.196–2.687		1.800	1.176–2.755	
cT	T0	1		<0.0001	1		<0.0001
	T1	1.964	0.268–14.411		2.529	0.343–18.643	
	T2	3.433	0.480–24.533		5.244	0.729–37.695	
	T3	4.568	0.638–32.695		6.761	0.940–48.619	
	T4	15.026	1.892–119.319		27.911	3.446–226.066	
	T4a	15.547	0.618–49.833		10.600	1.138–98.751	
cN	N0	1		0.0147	1		0.1544
	N+	1.333	1.058–1.678		1.201	0.933–1.547	
Year of diagnosis	≤2006	1		<0.0001	1		<0.0001
	2006–2009	0.503	0.396–0.638		0.556	0.424–0.728	
	>2009	0.623	0.350–1.108		0.933	0.503–1.731	

**Figure 2:**
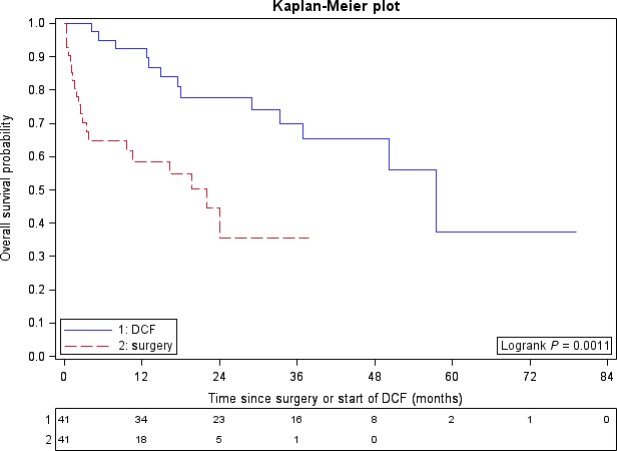
Overall survival according to Docetaxel, cisplatin, and 5‐fluorouracil (DCF) and surgery among matched sample (*n* = 82).

For the matched‐pair analysis, 20 and 13 deaths were observed in the surgery group and DCF group, respectively. Median OS was 22 months and 57 months for the surgery group and DCF group, respectively (log‐rank *P* = 0.0011) (Fig. 2). In Cox multivariate analysis, the DCF group was associated with favorable OS compared with the surgery group (HR = 0.29; 95% IC, 0.14–0.64; *P* = 0.0019) (Table 3).

**Table 3:** Cox regression for the matched‐pair analysis (*n* = 82)

		Univariate Cox analysis			Multivariate Cox analysis		
Parameters		HR	IC95%	*P*	HR	IC95%	*P*
Treatment	Surgery alone	1		0.0019	1		0.0019
	DCF	0.297	0.138–0.640		0.293	0.135–0.636	
Age	≤55	1		0.0925	1		0.0932
	55–65	2.363	1.083–5.158		2.354	1.072–5.167	
	>65	1.717	0.655–4.504		1.818	0.689–4.793	
Gender	Men	1		0.9903			
	Women	1.005	0.435–2.323				
Localization	Gastroesophageal junction and lower third of esophagus	1		0.8876			
	stomach	1.051	0.529–2.085				
Signet ring cell	No	1		0.4751			
	Yes	1.478	0.506–4.322				
cT	T1	1		0.2989			
	T2	1014628					
	T3	1601500					
	T4	9659415					
	T4a	1068538					
cN	N0	1		0.2067			
	N+	1.645	0.760–3.560				
Year of diagnosis	≤2006	1		0.7410			
	2006–2009	0.679	0.219–2.105				
	>2009	0.893	0.186–4.292				

### Surgical results

The type of surgery, the extent of resection, and the pathologic tumor stage and nodal status for the observational dataset are described in the Table 4. The incidence of postoperative morbidity was 52% in surgery group and 34% in the DCF group. Major complications (Dindo‐Clavien grade 3–5) arose in nine patients (14.5%) in the DCF group, and 89 patients (11.2%) in the surgery group (*P* = 0.57). The incidence of postoperative mortality was 3.2% in the DCF group and 2.9% in surgery group Table 4.

**Table 4:** Surgical characteristics in the observational dataset

	Patients with surgery only		DCF Patients		*P* value
	*n* = 789		*n* = 62		
	*n*	%	*n*	%	
Surgical procedure					<0.001
Subtotal gastrectomy	273	34.82	7	11.48	
Total gastrectomy	364	46.43	37	60.66	
Lewis‐Santi esophagectomy	147	18.75	17	27.87	
Missing	5		1		
Lymphadenectomy					1
Yes	613	95.93	47	95.92	
No	26	4.07	2	4.08	
Missing	150		13		
Lymphadenectomy extent					0.1391
D1	206	33.66	10	22.22	
>D1	406	66.34	35	77.78	
Missing					
Major surgical complications (grade 3–5 Dindo‐Clavien)					0.5741
Yes	89	11.2	9	14.5	
No	700	88.7	53	85.5	
Resection extent					0.7801
R0	719	91.48	56	90.32	
R1	54	6.87	5	8.06	
R2	13	1.65	1	1.61	
Missing	3		0		
Ratio of number of invaded lymph nodes on number of dissected lymph nodes					0.1413
<0.20 (Q3)	550	74.22	33	64.71	
≥0.20	191	25.78	18	35.29	
Missing	48		11		
pT					0.0190
T0	12	1.54	4	7.02	
T1	220	28.24	8	14.04	
T2	279	35.82	26	45.61	
T3	194	24.90	15	26.32	
T4	56	7.19	4	7.02	
In Situ	18	2.31	0	0	
missing	10		5		
pN					0.0660
N0	410	52.63	21	37.50	
N1	211	27.09	21	37.50	
N2	106	13.61	7	12.50	
N3	52	6.68	7	12.50	
Missing	10		16		

The characteristics of surgery for the matched‐pair population are described in the Table 5. Major complications (Dindo‐Clavien grade 3–5) arose in six patients (14.63%) in the DCF group and seven patients (17.07%) in the surgery group (*P* = 1). In the matched‐pair analysis, R0 resection rate was 85% in surgery group and 93% in the DCF group (*P* = 0.48).

### Compliance to DCF regimen

Among the 62 patients, 25 (40%) patients received three or more preoperative and postoperative DCF cycles, Table 5.

**Table 5:** Surgical characteristics in the matched‐pair population

	Patients with surgery only		DCF Patients		*P* value
	*n* = 41		*n* = 41		
	*n*	%	*n*	%	
Surgical procedure					0.2096
Subtotal gastrectomy	7	17.95	6	15.00	
Total gastrectomy	17	43.59	25	62.50	
Lewis‐Santi esophagectomy	15	38.46	9	22.50	
Missing	2		1		
Lymphadenectomy					1
Yes	23	95.83	31	96.88	
No	1	4.17	1	3.13	
Missing	17		9		
Lymphadenectomy extent					0.3483
D1	8	34.78	6	20.69	
>D1	15	65.22	23	79.31	
Missing			2		
Resection extent					0.4821
R0	35	85.37	38	92.68	
R1	5	12.20	3	7.32	
R2	1	2.44	0	0	
Major surgical complications (grade 3–5 Dindo‐Clavien)					1
Yes	6	14.63	7	17.07	
No	35	85.37	34	82.9	
Ratio of number of invaded lymph nodes on number of dissected lymph nodes					1
<0.20	23	66.67	22	68.75	
≥0.20	13	33.33	10	31.25	
Missing	2		9		
pT					0.0294
T1	0	0	3	8.11	
T2	12	30.77	4	10.81	
T3	13	33.33	21	56.76	
T4	12	30.77	8	21.62	
T4a	2	5.13	1	2.70	
Missing	2		4		
pN					1
N0	17	43.59	15	41.67	
N1	12	30.77	12	33.33	
N2	5	12.82	5	13.89	
N3	5	12.82	4	11.11	
Missing	2		5		

## Discussion

This study is the first head‐to‐head comparison between DCF regimen and surgery alone in resectable GEA. Being in the context of a retrospective study, we used propensity score analysis, a method designed to eliminate the bias caused by measured patient characteristics that affect both treatment and outcomes.

We showed a survival benefit with the use of DCF perioperative regimen with a HR of 0.29 (95% IC, 0.14–0.64) in the matched‐pair analysis and 0.59 (95% CI, 0.45–0.78) in the IPTW analysis. The consistency of these two analyses strengthens our conclusions. In the large phase III MAGIC trial, the HR for OS with ECF regimen was 0.75 (95% CI, 0.60–0.93) compared to surgery 1. In the phase III FFCD 9073 trial, the HR for OS with CF regimen was 0.69 (95% CI, 0.50–0.95) compared to surgery 2. Even though the improvement of R0 rate observed in DCF group was not statistically significant compared to surgery group, it is one of the highest reported in the literature (93%). In MAGIC and FFCD 9073 trials, R0 rates were 69% and 84%, respectively 1, 2. In 2012, Ferri et al. conducted a phase II single‐arm trial with DCF as perioperative chemotherapy in resectable GEA 5. In this study, 3‐year OS was 60%, which is comparable with our results (3‐year OS = 67%). In MAGIC and FFCD 9073 trials, 3‐year OS was 45% and 50%, respectively 1, 2. These results suggest the potential additional benefit of docetaxel in perioperative setting in terms of OS.

The DCF regimen is generally considered to be a toxic regimen due to high rates of myelosuppression. In our study, the toxicities were not reported because of a high rate of missing data in the clinical records. However, compliance rate (40%) was comparable with that reported in the MAGIC trial (41.6%) and no treatment‐related death was observed 1.

Other docetaxel‐containing perioperative regimens were assessed in phase II trials in resectable GEA. They demonstrated the feasibility of these regimens, and a high R0 rate (90–96%) 7, 8. Pathological complete responses (pCR) were 10–17% in these taxane‐based regimens 5, 7, 8, 9, 10. In our study, the pCR was lower than these trials (7%). However, it was higher than surgery group (1.4%), as well as MAGIC ECF protocol (no pCR reported) and FFCD 9703 CF protocol (3%) 1, 2. Previous reports confirmed the pCR rate as an independent prognostic factor of OS in GEA patients 11, 12, 13, 14.

In our study, postoperative morbidity and mortality were observed in 14.5% and 3.2% of the patients in DCF group. Even though these rates are slightly higher than 10% of morbidity and 0% of mortality reported by Ferri et al. in selected patients, they are similar to S group 5.

Our study does have other limitations. First, sample size of DCF arm was only 62. Then, as it is a retrospective study and even though different validated statistically analyses were applied to eliminate differences between two groups, uncontrolled biases are still possible and PS method cannot provide the level of evidence of randomized trials. However, the efficacy of DCF regimen is concordant to previous results observed in different phase II trials. Then, adverse events of DCF regimen could not be estimated.

The neoadjuvant approach with docetaxel‐based chemotherapy continues to be investigated in prospective randomized trials. The German AIO phase II/III FLOT4 study randomized 714 patients with resectable GEA either to the standard six cycles of perioperative ECF or to four cycles of 5‐FU, leucovorin, oxaliplatin, and docetaxel (FLOT) preoperatively and four cycles of FLOT postoperatively. Phase II data presented at the 2015 ASCO Annual Meeting showed that pCR rates were 12.8% with FLOT versus 5.1% with ECF. Korean investigators are combining docetaxel, oxaliplatin, and S‐1 as neoadjuvant therapy in addition to standard S‐1 adjuvant therapy for resectable but locally advanced GEA (T2–3/N+ or T4/N either +/‐) in the PRODIGY trial. Finally, the German NEO‐FLOT trial mirrors the PRODIGY trial, but uses 5‐FU and leucovorin instead of S‐1, with slightly lower doses of oxaliplatin. The results of all three trials are awaited to see whether newer combinations bring superior efficacy with tolerable toxicity.

## Conclusion

In conclusion, this population‐based study showed that perioperative DCF chemotherapy in resectable GEA is superior to surgery alone in terms of survival. A randomized phase III trial is needed to compare DCF to standard ECF or CF regimens to investigate the potential survival benefit of docetaxel in perioperative setting in resectable GEA. Future trials should also include a quality‐of‐life analysis to evaluate the clinical benefit between these regimens.

## Ethical Statements

All procedures followed were in accordance with the ethical standards of the responsible committee on human experimentation (institutional and national) and with the Helsinki Declaration of 1964 and later versions. Informed consent or substitute for it was obtained from all patients for being included in the study.

## Conflict of Interests

The authors declare that they have no competing interests.

## Supporting information


**Table S1**. Multivariate logistic regression to estimate the propensity score
