# Supplementary material for: Docetaxel, Cisplatin, and 5‐Fluorouracil as perioperative chemotherapy compared with surgery alone for resectable gastroesophageal adenocarcinoma
Source: Cancer Med. 2016 Oct 11;5(11):3085–93. doi: 10.1002/cam4.885 (PMC5119963; doi:10.1002/cam4.885)
Supplement: Supplementary file 1 — Table S1. Multivariate logistic regression to estimate the propensity score [file CAM4-5-3085-s001.doc]

**Supplementary table 1.** Multivariate logistic regression to estimate the propensity score

| **Variables** | | **OR** | **CI 95%** | **p value** |
| --- | --- | --- | --- | --- |
| **Age** | **≤65** | 1 |  | <0.0001 |
|  | **65-75** | 0.352 | (0.141-0.880) |  |
|  | **>75** | 0.044 | (0.014-0.141) |  |
| **Sex** | **Men** | 1 |  | 0.9315 |
|  | **Women** | 0.961 | (0.387-2.386) |  |
| **Localisation** | **esogastric junction and lower third of esophagus** | 1 |  | 0.0399 |
|  | **Stomach** | 0.406 | (0.172-0.959) |  |
| **Signet ring cell** | **No** | 1 |  | 0.5487 |
|  | **Yes** | 1.514 | (0.390-5.870) |  |
| **cT** | **T0, T1** | 1 |  | 0.0001 |
|  | **T2, T3** | 35.994 | (6.475-200.095) |  |
|  | **T4** | 126.137 | (8.500->999.999) |  |
| **cN** | **N0** | 1 |  | 0.0575 |
|  | **N+** | 2.151 | (0.976-4.740) |  |
| **Year of diagnosis** | **≤2006** | 1 |  | <0.0001 |
|  | **2006-2006** | 22.566 | (6.221-81.851) |  |
|  | **>2009** | 358.325 | (62.690->999.999) |  |
